# Hospitalisations and length of stays in women with endometriosis: a data linkage prospective cohort study

**DOI:** 10.1016/j.eclinm.2024.103030

**Published:** 2025-01-15

**Authors:** Dereje G. Gete, Annette J. Dobson, Grant W. Montgomery, Mohammad R. Baneshi, Jenny Doust, Gita D. Mishra

**Affiliations:** a Australian Women and Girls' Health Research Centre, School of Public Health, Faculty of Medicine, The University of Queensland, Brisbane, QLD, 4006, Australia; b Institute for Molecular Bioscience, The University of Queensland, Brisbane, QLD, 4072, Australia

**Keywords:** Endometriosis, Hospitalisation, Length of stay, Healthcare utilisation, Data linkage

## Abstract

**Background:**

Women with endometriosis have more hospitalisations compared to those without the condition. However, no longitudinal study has examined hospital admission rates and lengths of stay before and after diagnosis. We examined all-cause hospital admissions and lengths of stay among women with, versus without, endometriosis, and before, versus after, diagnosis.

**Methods:**

This study included 13,501 women of reproductive age, born in 1973–78. The Australian Longitudinal Study on Women's Health survey data linked to administrative health records was used to identify women with endometriosis. Hospital admission rates and length of stays were examined using hospital records of patients admitted up to 2022. Analysis was conducted using mixed-effects zero-inflated negative binomial models.

**Findings:**

Women with endometriosis were more likely to be admitted to hospitals compared to those without the condition, with an adjusted incidence rate ratio (IRR) of 2.11 (95% CI: 1.83–2.43) for admissions per year. However, they had shorter hospital stays (IRR: 0.90; 0.81–0.99) for days per year and were more often discharged on the same day (odds ratio: 1.27; 1.20–1.33). Post-diagnosis, women experienced more hospitalisations and more days in hospital compared to their pre-diagnosis (IRR: 1.52; 1.22–1.88) and (IRR: 1.81; 1.53–2.14), respectively. Consistent findings were found for women with surgically confirmed or clinically suspected endometriosis.

**Interpretation:**

The higher number of hospitalisations among women with endometriosis, compared to those without, highlights the substantial burden of the condition on healthcare utilisation. The persistent frequent hospitalisations and longer stays post-diagnosis indicate recurrent endometriosis, posing significant management challenges.

**Funding:**

The Australian Longitudinal Study on Women's Health is funded by the Australian Government 10.13039/501100003921Department of Health and Aged Care. GDM and GWM are Australian 10.13039/501100000925National Health and Medical Research Council Leadership Fellows (GNT2009577 and GNT1177194).

**Research in context**

**Evidence before this study**

We conducted a search on PubMed for articles published in English up to June 18, 2024, using the following terms (title or abstract): Endometriosis OR ‘pelvic pain’ OR ‘inflammation’ AND ‘hospitalisation’ OR ‘hospital admission’ OR ‘hospital readmission’ OR ‘length of stays’ OR ‘health service’ OR ‘healthcare utilisation’ OR ‘healthcare use’.

Women with endometriosis have more all-cause hospitalisations and use of other healthcare services than those without the condition. However, no longitudinal study has examined the rate of hospital admissions and length of stay before and after the diagnosis of endometriosis.

**Added value of this study**

This study is the first to examine hospitalisation trends among women with endometriosis, both before and after diagnosis, using a nationally representative cohort with 27 years of linked health data.

We found that women with endometriosis had more all-cause hospital admissions but shorter stays, often with same-day discharges, compared to those without the condition. Women with endometriosis experienced more frequent and prolonged hospitalisations after their diagnosis compared to before, with similar patterns observed for both surgically confirmed and clinically suspected cases.

**Implications of all the available evidence**

The sustained high number of hospitalisations and longer stays post-diagnosis indicate the ongoing burden on the women and health systems. Further research should investigate the underlying causes of these hospital admissions and prolonged stays to develop targeted interventions.

## Introduction

Endometriosis, a chronic and often debilitating condition, affects approximately 14% (around 1 in 7) of Australian women by age 44–49.^1^ Characterised by endometrial-like tissue growing outside the uterus,^2^ it causes a wide range of symptoms, mainly dysmenorrhea, heavy menstrual bleeding, fatigue, depression, anxiety, and bowel problems.^3^ This condition contributes to more hospitalisations, greater use of healthcare resources, and a significant reduction in the quality of life for affected women.^4^^,^^5^ Despite advancements in medical and surgical treatments, endometriosis remains a condition with a high rate of recurrence, posing substantial challenges for long-term management.^6^

Surgery for endometriosis is beneficial, especially for those with pain or infertility issues and when medication offers limited relief.^7^ Yet, the recurrence of endometriosis post-surgery is common, leading to an ongoing risk of further surgical interventions.^8^^,^^9^ This may be due to the regrowth of lesions or incomplete removal in initial surgeries.^10^ A retrospective claims study in the USA revealed that endometriosis patients faced a substantial risk of surgical complications, further surgeries, and hospital admissions following their initial therapeutic laparoscopy or hysterectomy.^11^

Endometriosis can lead to increased hospitalisations due to the severity of the symptoms, the need for surgical intervention, complications, and diagnostic challenges.^12^^,^^13^ In the USA in 2019, a retrospective cohort study documented this trend, showing greater all-cause hospitalisations in women with endometriosis.^14^ However, no longitudinal study has examined hospital admissions and length of stay before and after the diagnosis of endometriosis.

The current study investigated all-cause hospital admissions and lengths of stay in two analyses: (1) comparing women with endometriosis, versus those without, and (2) for the same women before and after being diagnosed with endometriosis. This analysis is essential for understanding how the diagnosis of endometriosis influences subsequent healthcare use.

## Methods

### Study design and participants

This study utilised data from the Australian Longitudinal Study on Women's Health (ALSWH) linked to administrative health records. The ALSWH, an ongoing, comprehensive, large-scale prospective cohort study, examines various factors affecting women's health and well-being. Initiated in 1996, it enrolled over 40,000 women across three age cohorts, randomly selected from the Australian universal health insurance database (Medicare), covering all Australian citizens and permanent residents. From 1996 to 2021, the study conducted nine surveys of the same women at three-year intervals, either online or through postal questionnaires. This study specifically used data from the ALSWH cohort born in 1973–78, who were 18–23 years old in 1996. Detailed information about ALSWH's design, recruitment methods, and responses has been previously published.^15^, ^16^, ^17^ The ALSWH study received approval from the Human Research Ethics Committees at the University of Queensland and the University of Newcastle, and informed consent was obtained from participants for each survey.

**Fig. 1:**
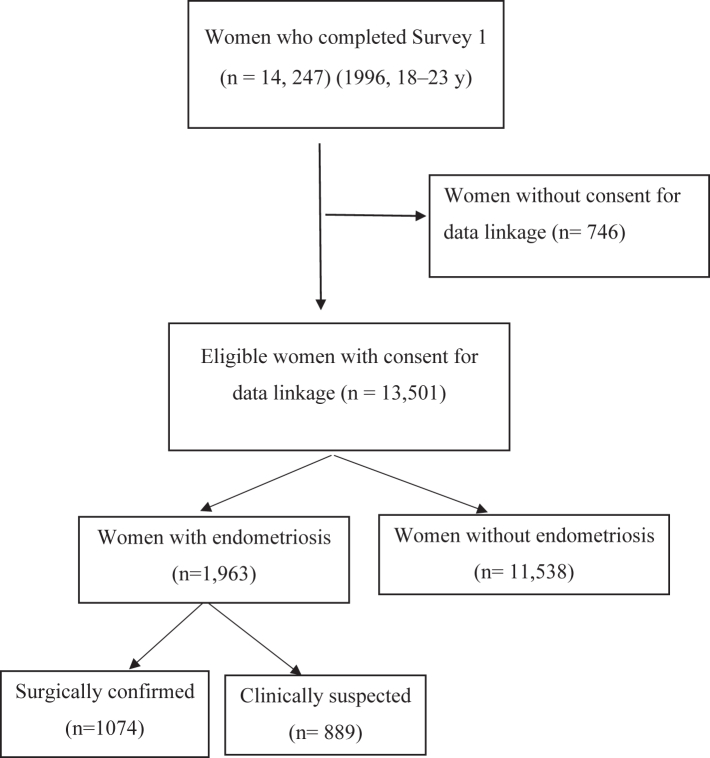
Flowchart of sample selection for comparing hospitalisations and length of stay between women with and without endometriosis.

In this study, data up to 2022 were included when the women were aged 43–49. Of the 14,247 initial participants in this cohort, approximately 95% (13,501) consented to link their survey data with administrative health records. Of these 13,501 women, 1963 women were diagnosed with endometriosis, while 11,538 were not as shown in Fig. 1.

### Assessments of endometriosis

Data on endometriosis were sourced from self-reported ALSWH surveys and administrative health records. The initial self-reported endometriosis occurred in the year 2000 at the second survey, where participants were asked, “Have you ever been told by a doctor that you have endometriosis?”

To identify women diagnosed with endometriosis, we linked the survey responses with three key administrative health databases: the Medicare Benefits Schedule (MBS), which lists healthcare services mainly doctor visits subsidised by Medicare; the Pharmaceutical Benefits Scheme (PBS), detailing medications dispensed and subsidised under Medicare; and the Admitted Patient Data Collections, which includes individual hospital separation records from public and some private hospitals. The hospital database recorded each admission using either the International Classification of Diseases, 9th Revision, Clinical Modification (ICD-9-CM) or 10th Revision, Australian Modification (ICD-10-AM). The PBS database was used to identify women prescribed medications typically restricted for endometriosis, including Goserelin (code: 01454M), Medroxyprogesterone 10 mg × 100 tablets (code: 02722G), and Nafarelin (code: 02962X). Although these medications are primarily used for endometriosis, they may also be prescribed for other conditions in Australia, such as prostate or breast cancer (Goserelin), menstrual disorders or contraception (Medroxyprogesterone), and as puberty blockers or in assisted reproductive technology (Nafarelin). However, Nafarelin is not listed on the PBS for use as a puberty blocker and is unlikely to have been prescribed for this purpose in the study cohort. Additionally, the PBS item number for Nafarelin prescribed in assisted reproductive technology differs from that used for endometriosis. To ensure accuracy, we relied on prescription item numbers specific to endometriosis. The PBS data were then combined with self-reported survey responses of physician-diagnosed endometriosis for analyses. This approach reduces the risk of misclassification and enhances the validity of endometriosis identification in our study. The methods for determining endometriosis cases and the specific identification codes used in each database are detailed in other publications^1^ and summarised in Supplementary Table S1.

In our analysis, we differentiated endometriosis cases into two categories based on information from these linked data sources: surgically confirmed and clinically suspected cases. Surgically confirmed cases were identified through records in the MBS or hospital databases, where a diagnosis of endometriosis was explicitly recorded. Conversely, women who self-reported endometriosis in the ALSWH surveys or had been prescribed medications restricted for endometriosis (as indicated by the PBS database) without hospital or procedure codes that indicated a surgical diagnosis of endometriosis were categorised as clinically suspected cases.

### Assessments of hospitalisations and length of stays

All-cause hospitalisations and length of stays were examined using admitted patient hospital records from 1996 to 2022. The admitted patient hospital database includes records for all patient separations from public hospitals in all states and territories, and all private hospitals in New South Wales, Queensland, Victoria, and Western Australia. A ‘separation’ denotes a complete care episode for an admitted patient, encompassing the entire hospital stay from admission to discharge, transfer, or death.

The length of stay of a patient for an episode of admitted patient hospital care was measured in days, calculated as the difference between the admission and separation dates, deducting total leave days, with the admission day counted as the first day. Hence, if a patient's admission and discharge occur on the same day, the length of stay was considered one day.

### Assessments of women's characteristics

This study evaluated the baseline characteristics of women using self-reported data from the ALSWH surveys, including women's age, residential area, state or territory, education, marital status, ability to manage their income, smoking habits, alcohol consumption, body mass index (BMI), physical activity, and parity. Data on private health insurance were obtained from the admitted patients' hospital records at their most recent hospitalisations.

A woman's age at the time of admission was determined by subtracting her date of birth in years from the admission date (hospital records), divided by 365.25. Residential areas were categorised as major cities, inner regional, outer regional, and remote areas.^18^ Education was categorised as up to year 12 or equivalent, trade/apprenticeship/certificate/diploma, and university/higher degree. Smoking status was classified as never-smoker, ex-smoker, or current-smoker.^19^ Alcohol consumption was categorised into four groups: non-drinkers, low-risk drinkers (up to 14 drinks/week), risky drinkers (15–28 drinks/week), and high-risk drinkers (more than 28 drinks/week).^20^ However, due to the small number of women who were high-risk drinkers (n = 83, 0.6%), this category was merged with the risky drinker group. BMI categories were underweight (BMI less than 18.5 kg/m^2^), normal weight (BMI 18.5 to less than 25 kg/m^2^), overweight (BMI 25 to less than 30 kg/m^2^), and obese (BMI 30 kg/m^2^ or more). Physical activity was evaluated using metabolic equivalent (MET) values, calculated from the frequency and duration of walking, moderate, and vigorous activities, and categorised into four levels: sedentary (less than 40 MET minutes/week), low (40 to less than 600 MET minutes/week), moderate (600–1200 MET minutes/week), and high (1200 MET minutes/week or more).^21^

### Statistics

Baseline characteristics of women with and without endometriosis (including both surgically confirmed and clinically suspected cases) were summarised by percentages (%) for categorical variables and mean (SD) for continuous variables and compared using Pearson's chi-square tests and t-tests., Due to skewed distributions, all-cause hospitalisations were categorised as none, same-day, and more than one day, and expressed as percentages (%). The median (quartiles) number of days was provided for hospitalisations of more than one day.

The observation times for hospitalisation varied among the States and Territories (Supplementary Table S2) so the hospital data were analysed as numbers of admissions per person-year at risk or length of stays (days) per person-year with hospital admission. Hospitalisations were classified as same-day admissions or admissions of two or more days (overnight admissions). All the data were very skewed with an excess of zeros; therefore, zero-inflated negative binomial models using the ‘glmmTMB’ package in R software were used, with random effects to account for the multiple years of observation contributed by each woman and offset terms to account for differences in observation periods between States and Territories. Results were described using incidence rate ratios (IRR) with 95% confidence intervals (CIs) for the number of hospital admissions or number of days in hospital per year and odds ratios (ORs) with 95% CIs were reported for hospitalisation compared to no hospitalisation in a year.

Several comparisons were made between women with or without a diagnosis of endometriosis, women with surgically confirmed versus clinically suspected endometriosis, and women before and after the diagnosis. For women diagnosed with endometriosis, the year of first diagnosis was identified using all four data sources (ALSWH surveys, MBS, PBS, and hospital admissions). The number of hospitalisations and length of stay in the year of diagnosis was compared with the years before and after diagnosis.

All analyses included adjustments for age at admissions, area of residence, educational background, and ability to manage available income. These variables were chosen based on their known associations with endometriosis diagnosis and hospitalisation patterns and were further assessed by the study participants. Educational data were sourced from ALSWH Survey 3 (conducted when participants were aged 25–30), ensuring participants were likely to have completed higher education. Information on income and area of residence was obtained from the 1996 baseline survey. Statistical analysis was conducted using R software version 4.2.3 and Stata software version 18 (StataCorp, College Station, TX).

### Ethics

The ALSWH was originally approved by the Human and Research Ethics Committees (HREC) of the University of the Newcastle (UoN) in 1995 (Clearance number: H 076 0795) and by The University of Queensland (UQ) in 2004 (reference number: 2004000224). The most recent amendment for ALSWH Survey 8 of the 1973–78 cohort was approved by UoN HREC on 8 November 2017 and ratified by UQ HREC on 9 November 2017. Ethical approval for data linkage was obtained from the UoN HREC on 31 January 2012 (Clearance Number: H-2011-0371) and from the UQ HREC on 9 February 2012 (Number: 2012000132).

### Role of funding source

The funders had no role in the study design, data collection, data analysis, data interpretation, or writing of the report.

## Results

The study analysed data from 13,501 women born between 1973 and 1978, over the period from 1996 to 2022 (Fig. 1). We found a 14.5% (1963) prevalence of endometriosis by age 49, with 7.9% (1074) surgically confirmed and 6.6% (889) clinically suspected cases. The mean age of women at diagnosis of endometriosis was 33 (SD 7.6).

We observed a higher percentage of endometriosis among women living in major urban areas, those who were married, underweight, and consumed alcohol (Table 1). Parous women were less likely to have a diagnosis of endometriosis. A higher proportion of endometriosis patients had private health insurance.

**Table 1:** Baseline characteristics in 1996 for women with and without endometriosis.^a^

Women's characteristics	All women (n = 13,501)	Endometriosis		P-value^b^
		Yes (n = 1963)	No (n = 11,538)	
Woman's age (years), mean (SD)	20.7 (1.5)	20.7 (1.5)	20.6 (1.5)	0.10
Area of residence (%)^c^				0.09
Major cities	6996 (51.8)	14.4	85.6	
Inner regional	4070 (30.2)	15.3	84.7	
Outer regional	1986 (14.7)	14.4	85.6	
Remote	440 (3.3)	10.5	89.5	
States and Territories (%)				0.06
New South Wales	3673 (27.2)	14.3	85.7	
Victoria	3621 (26.8)	15.8	84.2	
Queensland	3009 (22.3)	14.8	85.2	
South Australia	1005 (7.4)	12.3	87.6	
Western Australia	1476 (10.9)	14.3	85.7	
Tasmania	372 (2.8)	12.4	87.6	
Northern Territory	130 (1.0)	13.1	86.9	
Australian Capital Territory	215 (1.5)	10.7	89.3	
Marital status (%)^c^				0.04
Married	1204 (8.9)	16.8	83.2	
De facto/separated/divorced	1970 (14.6)	15.0	85.0	
Single	10,262 (76.0)	14.2	85.8	
Educational status (%)^c^				0.66
Up to year 12 or equivalent	9529 (70.6)	14.6	85.4	
Trade/apprenticeship/certificate/diploma	2401 (17.8)	14.7	85.3	
University/higher degree	1494 (11.1)	13.8	86.2	
Ability to manage income (%)^c^				0.57
It is impossible/difficult all the time	2498 (18.5)	14.5	85.5	
It is difficult some of the time	4464 (33.1)	14.3	85.7	
It is not too bad	4787 (35.5)	14.6	85.4	
It is easy	1703 (12.6)	14.9	85.1	
Smoking status (%)^c^				0.05
Never smoked	6728 (49.8)	14.8	85.2	
Ex-smoker	1976 (14.6)	16.0	84.0	
Current smoker	4216 (31.2)	13.7	86.3	
Alcohol intake (%)^c^				0.05
Non-drinker	1188 (8.8)	13.2	86.8	
Rarely drinker	4590 (34.0)	15.6	84.4	
Low-risk drinker	6844 (50.7)	14.3	85.7	
Risky drinker	730 (5.4)	13.0	87.0	
Physical activity (%)^c^				0.45
Sedentary, <40 MET min/week	842 (6.2)	17.8	82.2	
Low, 40 to <600 MET min/week	2872 (21.3)	16.5	83.5	
Moderate, 600 to <1200 MET min/week	2090 (15.5)	17.0	83.0	
High, ≥1200 MET min/week	3089 (22.9)	15.8	84.2	
Body mass index (%)^c^				0.01
Underweight, <18.5 kg/m^2^	1147 (8.5)	16.5	83.5	
Healthy weight, 18.5 to <25 kg/m^2^	8035 (59.5)	15.1	84.9	
Overweight, 25 to <30 kg/m^2^	1817 (13.5)	14.3	85.7	
Obese, ≥30 kg/m^2^	761 (5.6)	11.2	88.8	
Parity (%)^c^				0.17
Nulliparous	11,925 (88.3)	14.7	85.3	
Parous	1313 (9.7)	13.3	86.7	
Private health insurance^d^				<0.0001
Yes	6211 (46.0)	17.8	82.2	
No	4939 (36.6)	12.8	87.2	
Unknown/not stated	618 (4.6)	17.2	82.8	

a Values are mean (SD) or percentage (%). Baseline characteristics of women were presented as row percentages for those with or without endometriosis and as column percentages for all women.

b P-values from Pearson chi-square or t-tests.

c Missing values (area of residence: n = 9, marital status: n = 65, education: n = 69, manage on income: n = 49, smoking status: n = 581, alcohol intake: n = 149, physical activity: n = 4608, BMI: n = 1741, parity: n = 263).

d Information on private health insurance was obtained from the admitted patients' hospital database at their most recent hospitalisations (n = 11,768). Endometriosis was identified using four data sources (ALSWH survey, MBS, Hospital, and PBS databases) up to 2022.

The descriptive analysis presented in Table 2 shows that women with endometriosis experienced more all-cause hospital admissions but had shorter hospital stays compared to those without. When excluding those with no hospitalisations, the median length of hospital stay per year for women with endometriosis was shorter than for those without the condition, with a median (Q1, Q3) of 1 day (0, 4) versus 2 days (0, 4), respectively. Additionally, after being diagnosed, women with endometriosis had more hospitalisations and longer hospital stays than before their diagnosis (Supplementary Table S3).

**Table 2:** All causes of hospitalisations and length of stays according to endometriosis categories up to 2022.^a^

Hospitalisations and length of stays	All women	All endometriosis	Subgroups of endometriosis		
			Surgically confirmed	Clinically suspected	No endometriosis
Number of women	13,501	1963	1074	889	11,538
Number of all observations	287,115	41,845	23,059	18,786	245,270
Hospitalisations among all observation					
No hospitalisation (%)	81.5	74.5	70.3	79.6	82.7
Same-day hospitalisation (%)	13.2	16.8	19.3	13.6	12.6
Two or more days (%)	5.3	8.7	10.3	6.7	4.7
Number of observations for same-day and two or more days	52,983	10,667	6841	3826	42,316
Length of stay (days per year)					
Mean (SD)	3.2 (9.7)	2.9 (6.7)	2.8 (6.5)	3.1 (6.9)	3.3 (10.3)
Median (Q1, Q3)	1 (0, 4)	1 (0, 4)	1 (0, 4)	1 (0, 4)	2 (0, 4)

Endometriosis was identified using four data sources (ALSWH survey, MBS, Hospital, and PBS databases). Surgically confirmed cases were identified using records from the MBS or hospital databases. Clinically suspected cases included women who either reported endometriosis in the ALSWH surveys or were prescribed medications specific to endometriosis as indicated by the PBS database.

a Values are column percentage (%), mean (SD), and median (quartiles).

Analysing the data from hospital-admitted patients, pelvic peritoneal endometriosis was the most common diagnosis, accounting for 50.5% of principal and 32.3% of additional diagnoses, as shown in Supplementary Figure S1. Uterine endometriosis came next, comprising 20.1% of principal and 19.9% of additional diagnoses, followed by ovarian endometriosis (13.2% of primary and 17.1% of secondary diagnoses).

**Fig. 2:**
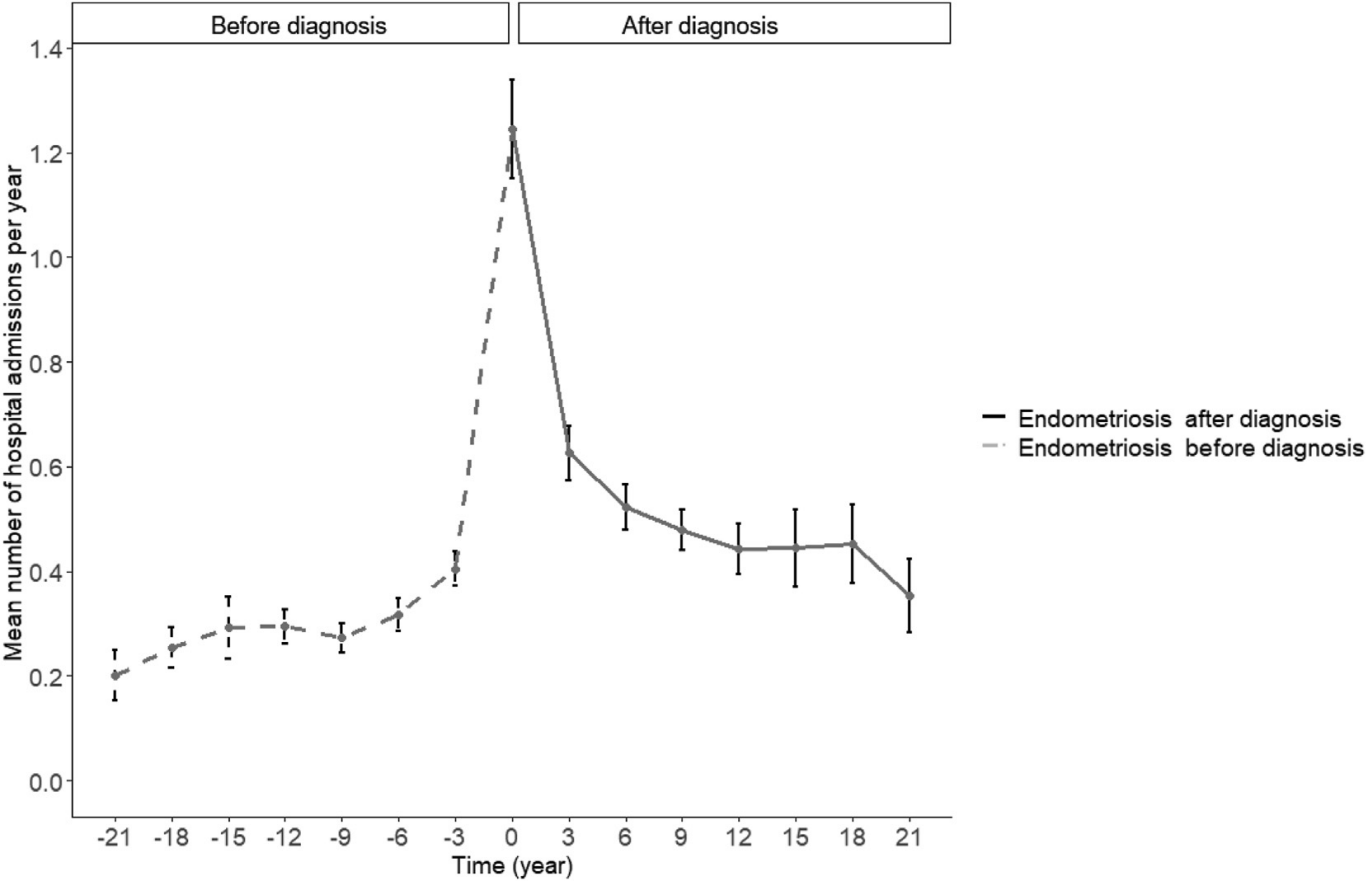
Mean number of hospital admissions according to time of diagnosis among women with endometriosis (n = 1963).

**Fig. 3:**
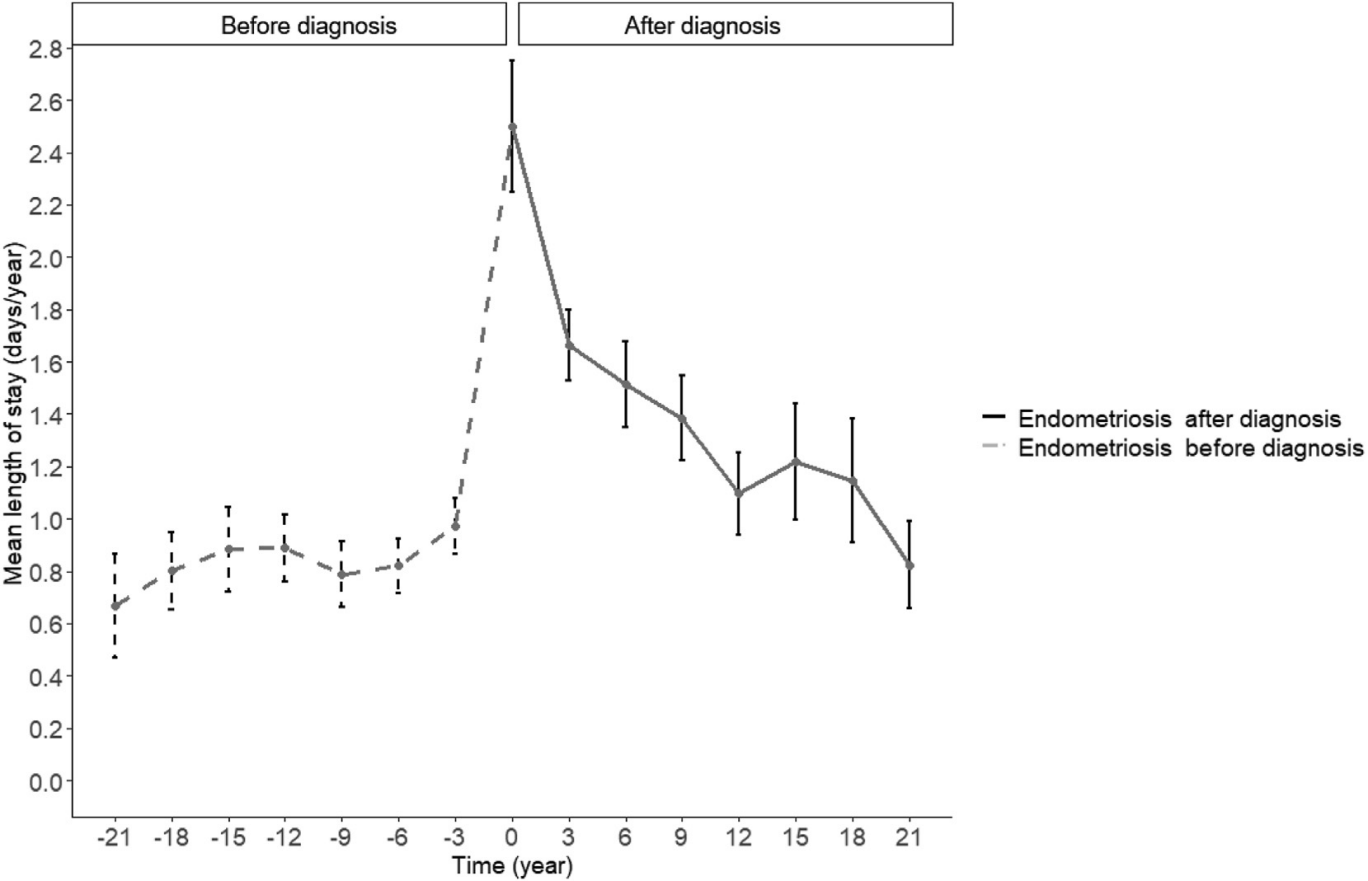
The mean length of hospital stays according to time of diagnosis among women with endometriosis (n = 1963).

The average number of hospitalisations per year and the number of days in hospital per year were stable in the early years, slightly increased before the diagnosis, and spiked around the time of the endometriosis diagnosis. After the diagnosis, they decreased immediately but remained at a higher level than before the diagnosis (Figs. 2 and 3). This trend was consistent regardless of whether endometriosis was surgically confirmed or clinically suspected, although surgically confirmed cases experienced a marginally higher mean number of hospital stays and admissions (Supplementary Figures S2 and S3).

**Fig. 4:**
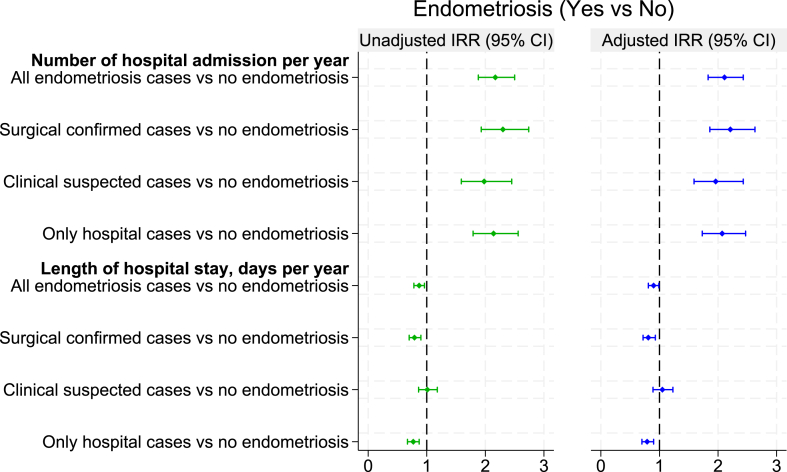
Incidence Rate Ratio of the number of all-cause hospitalisations and length of stay among women with or without endometriosis, n = 13,501 (endometriosis cases, n = 1963 versus no endometriosis, n = 11,538).

Compared to those without endometriosis, women with endometriosis had a higher number of all-cause hospital admissions per year after adjusting for age, area of residence, education, and income, with adjusted incidence rate ratios (IRR) of 2.11 (95% CI: 1.83–2.43) (Fig. 4; Supplementary Table S4). Consistent evidence was observed whether endometriosis was surgically confirmed, 2.21 (1.86–2.63) or clinically suspected 1.96 (1.59–2.43). Despite the higher frequency of admissions, women diagnosed with endometriosis had fewer total days in hospital per year compared to those without the condition, with an IRR of 0.90 (0.81–0.99). We also observed similar findings among only hospital-admitted patients, showing that women with endometriosis experienced more hospitalisations 2.07 (1.73–2.47) but had shorter hospital stays than those without the condition 0.79 (0.70–0.90).

Further analysis was conducted, specifically focusing on individuals with hospitalisations, i.e., excluding those without any hospitalisations, to examine same-day hospitalisations. We found that women with endometriosis were more likely to be discharged on the same day, with an adjusted odds ratio for same-day hospitalisation of 1.27 (95% CI: 1.20–1.33) (Supplementary Table S4). Similar evidence was found regardless of surgical confirmation or clinical suspicion of endometriosis.

This study also compares the IRR of hospitalisations before and after the diagnosis of endometriosis, focusing exclusively on women with endometriosis. Women with endometriosis experienced higher all-cause hospitalisations and longer hospital stays after the diagnosis compared to before the diagnosis, 1.52 (1.22–1.88) and 1.81 (1.53–2.14), respectively (Table 3).

**Table 3:** The incidence rate ratio of the hospital admissions and length of stay before and after diagnosis of endometriosis, n = 1963.

Outcomes	Time of endometriosis diagnosis	Unadjusted model (95% CI)	Adjusted model (95% CI)^a^
Number of hospital admissions per year	**Before diagnosis**	1.00	1.00
	**During diagnosis**		
	IRR for more hospitalisations	1.91 (1.56–2.34)	1.86 (1.50–2.30)
	OR for no hospitalisations	0.13 (0.12–0.15)	0.08 (0.07–0.09)
	**After diagnosis**		
	IRR for more hospitalisations	1.56 (1.33–2.34)	1.52 (1.22–1.88)
	OR for no hospitalisations	0.69 (0.65–0.72)	0.48 (0.44–0.53)
Length of stay (days/year)	**Before diagnosis**	1.00	1.00
	**During diagnosis**		
	IRR for more hospital days	0.84 (0.71–0.99)	1.08 (0.91–1.27)
	OR for no hospital days	0.13 (0.12–0.15)	0.08 (0.07–0.09)
	**After diagnosis**		
	IRR for more hospital days	1.21 (1.06–1.38)	1.81 (1.53–2.14)
	OR for no hospital stays	0.69 (0.65–0.72)	0.48 (0.44–0.53)

IRR, incidence rate ratio; ORs, odds ratios; CI, confidence interval. The time of diagnosis indicates whether hospitalisations occurred before or after the first diagnosis of endometriosis. Hospitalisation during diagnosis refers to the time when the patient was admitted to the hospital while the diagnosis of endometriosis was made. This analysis exclusively involved individuals with endometriosis and a group of women who had not yet been diagnosed with the condition (before diagnosis) were used as a reference group.

a Adjusted for age at admission, age at diagnosis, area of residence, education, and management income.

## Discussion

This nationwide prospective cohort study revealed that women with endometriosis had a significantly higher number of all-cause hospital admissions than those without the condition, regardless of whether the diagnosis was surgically confirmed or clinically suspected. However, these women had shorter hospital stays and were more often discharged on the same day. Women with endometriosis experienced more hospital admissions and longer hospital stays after their diagnosis compared to before.

Our findings of increased hospital admissions for women with endometriosis are consistent with the findings of Soliman et al., though their study was limited to a descriptive analysis.^14^ This increase could stem from the severity of the symptoms, complications, difficulties in diagnosis, and the substantial impact on the patient's quality of life.^12^^,^^13^ The higher rate of hospital admission following diagnosis may also result from the recurrent nature of the disease,^10^ post-surgical complications, and the requirement for ongoing treatment. Roman et al.^22^ showed that within a decade following initial surgery, up to 28% of patients might need a repeat surgical intervention, primarily due to the recurrence of pain or endometriosis, as well as for treating infertility issues. The persistence of microscopic endometriotic lesions, which remain undetectable during surgery, is a recognised contributor to disease recurrence.^6^^,^^10^ Vizziell et al.^23^ demonstrated that advanced imaging methods, such as three-dimensional laparoscopy and real-time near-infrared imaging with indocyanine green, enhance lesion detection but remain limited to identifying all microscopic foci. This highlights the critical need for adjunctive treatments and thorough postoperative care to manage the residual disease and reduce the risk of recurrence.

Women with endometriosis may require hospitalisation due to various factors, including chronic pelvic pain, infertility, and complications arising from deep infiltrating endometriosis.^24^, ^25^, ^26^, ^27^, ^28^, ^29^ Chronic pelvic pain, often linked to central sensitisation,^24^ typically requires comprehensive, multidisciplinary care. Infertility is another common cause, frequently necessitating hospitalisations for procedures such as diagnostic laparoscopy or assisted reproductive technologies.^25^ Severe intestinal or urinary stenosis caused by deep infiltrating endometriosis often requires surgical interventions to manage obstructions or alleviate symptoms.^26^^,^^27^ Moreover, comorbidities such as irritable bowel syndrome and mental disorders can intensify the disease burden,^28^^,^^29^ further contributing to increased hospitalisation rates.

Although this study highlights the higher number of all-cause hospitalisations in women with endometriosis, it did not examine the specific indications for these admissions. Endometriosis presents with a diverse range of symptoms and complications, including chronic pain, heavy menstrual bleeding, chronic fatigue, and bowel and urinary symptoms,^3^ which likely contributes to higher hospitalisation rates. These hospitalisations may result from diagnostic, surgical, or therapeutic interventions for endometriosis and the management of associated comorbidities. Future research should investigate the specific reasons for hospitalisations, including the contributions of endometriosis-related treatments and comorbidities, to better inform anticipatory care and healthcare resource planning.

In this study, women diagnosed with endometriosis had shorter hospital stays than those without the condition. This aligns with the Australian Institute of Health and Welfare report (2023),^1^ which used descriptive methods to analyse endometriosis-related hospitalisations among admitted patients. In the early stages of endometriosis, minimally invasive surgeries, such as laparoscopy, may have the potential to expedite recovery and reduce hospitalisations^30^ Additionally, effective self-administered pain management at home^31^ and a shift towards outpatient care reduces the need for extended hospital stays. However, after being diagnosed, these women might face more hospitalisations and prolonged hospital stays than before their diagnosis, mostly due to the need for surgical procedures, the aggravation of disease severity, and the management of associated conditions. Surgical treatments, frequently essential after diagnosing endometriosis, typically result in extended periods of hospital care. As the condition progresses, it often becomes more severe, with an escalation in complications that demand more extensive medical care. Moreover, the treatment of concurrent conditions, such as infertility or gastrointestinal issues, which may be identified after the initial diagnosis, contributes to more hospital admissions and longer hospital stays. A pooled cross-sectional study in the USA observed decreased inpatient admissions for endometriosis over the past decade.^32^ Conversely, the study noted an increase in the length of hospital stays, surgical complications, and related hospital costs. Focusing on descriptive analysis, the study examined national trends of inpatient admissions at three distinct time points and exclusively considered women with endometriosis as the primary diagnosis.

The major advantage of this study lies in its utilisation of a large, nationally representative prospective cohort study followed over 27 years. The study employed national administrative health records to examine the rate of hospitalisations and length of stay. The national administrative health records were also linked with self-reported data to identify cases of endometriosis. We investigated hospitalisation trends among women with both surgically confirmed and clinically suspected endometriosis. However, a limitation is that 45% of the women in the endometriosis group were clinically suspected cases without surgical confirmation. While this method aims to prevent diagnostic delays, it may overestimate the prevalence of endometriosis and increase the risk of misdiagnosis.^33^ Our study also acknowledges the absence of detailed information on the severity of endometriosis, which might influence hospitalisation rates. Despite this, we evaluated the location of endometriosis as a principal or additional diagnosis using the 4th character of the ICD-10-AM code in the hospital database, providing valuable insights for clinical management and treatment approaches. The higher prevalence of endometriosis among women in major urban areas may reflect detection bias due to improved access to specialist evaluations, surgical interventions, and regular medical screenings. A higher proportion of women with endometriosis had private health insurance than those without the condition. This difference may influence the study findings, as private health insurance often enables better access to diagnostic services, specialist care, and elective procedures, potentially resulting in higher detection rates and distinct management pathways for endometriosis.

The admitted hospital database includes records from all public hospitals nationwide and private hospitals in New South Wales, Queensland, Victoria, and Western Australia, collectively accounting for approximately 88% of Australia's population as of March 2024. Our study also demonstrated that 87% of participants resided in these states.^34^ This substantial coverage underscores the representativeness of our findings, providing a near-population-level denominator for both cases and controls. Case ascertainment was further strengthened by integrating self-reported survey data with MBS and PBS records, capturing diagnoses beyond hospital admissions.

Diagnosing and treating endometriosis presents significant challenges due to its complex, burdensome, and often recurrent nature. Women with endometriosis experience more all-cause hospitalisations compared to those without the condition, regardless of the diagnosis method. They also face higher hospital admissions and longer stays post-diagnosis, substantially impacting healthcare utilisation and costs. These findings highlight the need for improved management strategies and healthcare policies for women with endometriosis. Further research could explore the underlying causes of increased hospital admissions and longer hospital stays post-diagnosis to develop targeted interventions, as well as examine the effectiveness of post-surgery medical suppression therapy in reducing disease recurrence and hospitalisations.

## Contributors

DGG and GDM conceived and designed the study. DGG analysed the data and wrote the manuscript; AJD and GDM contributed to statistical methods. GDM, AJD, JD, GWM and MRB critically reviewed and revised the manuscript as needed. DGG, GDM, and AJD accessed and verified the underlying data of the study. All authors read and approved the final manuscript.

## Data sharing statement

The data underlying this article cannot be shared publicly for ethical reasons and the privacy protection of the individuals who participated in the study. The survey data will be shared upon reasonable request to the principal investigator (GDM). Access to linked health data requires approval from Human Research Ethics Committees and Data Custodians.

## Declaration of interests

We declare no competing interests.
