# Supplementary material for: Hospitalisations and length of stays in women with endometriosis: a data linkage prospective cohort study
Source: eClinicalMedicine. 2025 Jan 15;80:103030. doi: 10.1016/j.eclinm.2024.103030 (PMC11934863; doi:10.1016/j.eclinm.2024.103030)
Supplement: Supplementary Tables and Figures [file mmc1.docx]

**Supplement Contents**

| **Content** | **Page** |
| --- | --- |
| Supplementary Table 1: Administrative health data sources used to identify endometriosis | 2 |
| Supplementary Table 2: Years of availability for hospital data for women born in the 1973-78 cohort, by States/Territories | 3 |
| Supplementary Table 3: All-causes hospitalisations and length of stays according to time of endometriosis diagnosis | 4 |
| Supplementary Table 4: Incidence Rate Ratio of all-cause hospitalisations and length of stay among women with or without endometriosis (n=13501) | 5 |
| Supplementary Figure 1: Hospitalisations by site of endometriosis, 4th character ICD-10-AM code | 6 |
| Supplementary Figure 2: The mean number of hospital admissions according to time of diagnosis among women with surgically confirmed and clinically suspected endometriosis (n= 1963) | 7 |
| Supplementary Figure 3. The mean length of hospital stays according to time of diagnosis among women with surgically confirmed and clinically suspected endometriosis (n= 1963) | 8 |

| **Data source** | **Code(s)** | **Data available** |
| --- | --- | --- |
| MBS | 35641 | November 2000–November 2022 |
| PBS | Goserelin (code: 01454M)  Medroxyprogesterone 10 mg X 100 tablets (code: 02722G)  Nafarelin (code: 02962X) | October 2001–December 2022 |
| Admitted patient hospital data | International Statistical Classification of Diseases and Related Health Problems, 9th revision, clinical modification (ICD-9-CM) diagnostic codes 617·0–617·9  International Statistical Classification of Diseases and Related Health Problems, 10th revision, Australian modification (ICD-10-AM) diagnostic codes N80·0–N80·9 | January 1970–September 2022 |
| **Supplementary Table 1: Administrative health data sources used to identify endometriosis** | | |

| **State/Territory** | **Earliest record** | **Latest record** |
| --- | --- | --- |
| New South Wales | May 2001 | March 2021 |
| Victoria | July 2000 | December 2020 |
| Queensland | July 2002 | September 2022 |
| Western Australia | January 1970 | December 2021 |
| South Australia* | January 2000 | June 2020 |
| Tasmania* | January 2007 | December 2019 |
| Australian Capital Territory* | July 2004 | June 2020 |
| Northern Territory * | July 2000 | June 2019 |
| *Public hospital data only. | | |
| **Supplementary Table 2: Years of availability for hospital data for women born in the 1973-78 cohort, by States/Territories** | | |

| **Hospitalisations and length of stays** | **Time of endometriosis diagnosis (n= 1963)** | | |
| --- | --- | --- | --- |
|  | **Before diagnosis** | **During diagnosis** | **After diagnosis** |
| Number of all observations | 16,652 | 1613 | 23,580 |
| Hospitalisation among all observation  No hospitalisation (%)  Same-day hospitalisation (%)  Two or more days (%) | 80·0  13·9  6·1 | 34·6  40·4  25·1 | 73·3  17·2  9·5 |
| Number of observations for same-day and two or more days | 3324 | 1055 | 6288 |
| Length of stay (days per year)  Mean (SD)  Median (Q1, Q3) | - 1. (5·5)   1 (0, 4) | 1·9 (4·7)  1 (0, 2) | 3·2 (7·5)  1 (0, 4) |
| ^*^Values are column percentage (%), mean (SD), and median (quartiles). This analysis exclusively involved individuals with endometriosis (n= 1963). The time of diagnosis indicates whether hospitalisations occurred before or after the first diagnosis of endometriosis. Hospitalisation during diagnosis refers to the period when the patient was admitted to the hospital at the same time the diagnosis of endometriosis was made. | | | |
| **Supplementary Table 3: All-causes hospitalisations and length of stays according to time of endometriosis diagnosis^*^** | | | |

| **Outcomes** | **Women with endometriosis vs without endometriosis** | **Unadjusted model (95% CI)** | **Adjusted model (95% CI) *** |
| --- | --- | --- | --- |
| Number of hospital admissions per year | **No endometriosis** | 1.00 | 1.00 |
|  | **All endometriosis cases vs no endometriosis**  IRR for more hospitalisations  OR for no hospitalisations | 2·17 (1·88 – 2·50)  0·61 (0·59 – 0·62) | 2·11 (1·83 – 2·43)  0·54 (0·53 – 0·55) |
|  | **Surgically confirmed cases vs no endometriosis**  IRR for more hospitalisations  OR for no hospitalisations | 2·30 (1·93 – 2·74)  0·49 (0·48 – 0·51) | 2·21 (1·86 – 2·63)  0·41 (0·39 – 0·42) |
|  | **Clinically suspected cases vs no endometriosis**  IRR for more hospitalisations  OR for no hospitalisations | 1·98 (1·59 – 2·45)  0·82 (0·79 – 0·85) | 1·96 (1·59 – 2·43)  0·83 (0·80 – 0·87) |
|  | **Only hospital cases vs no endometriosis**  IRR for more hospitalisations  OR for no hospitalisations | 2·14 (1·79-2·56)  0·58 (0·56 – 0·60) | 2·07 (1·73 – 2·47)  0·51 (0·50 – 0·53) |
| Length of stay (days/ year)  First analysis ^†^ | **All endometriosis vs no endometriosis**  IRR for more hospital stays  OR for no hospitalisations | 0·87 (0·78 – 0·96)  0·61 (0·59 – 0·62) | 0·90 (0·81 – 0·99)  0·54 (0·53 – 0·55) |
|  | **Surgically confirmed cases vs no endometriosis**  IRR for more hospital stays  OR for no hospitalisations | 0·79 (0·70 – 0·90)  0·49 (0·48 – 0·51) | 0·81 (0·72 – 0·93)  0·41 (0·39 – 0·42) |
|  | **Clinically suspected cases vs no endometriosis**  IRR for more hospital stays  OR for no hospitalisations | 1.01 (0·86 – 1·18)  0·82 (0·79 – 0·85) | 1·05 (0·89 – 1·23)  0·83 (0·80 – 0·87) |
|  | **Only hospital cases vs no endometriosis**  IRR for more hospitalisations  OR for no hospitalisations | 0·77 (0·67 – 0·87)  0·58 (0·56 – 0·60) | 0·79 (0·70 – 0·90)  0·51 (0·50 – 0·53) |
| Length of stay (days/ year)  Second analysis ^‡^ | **All endometriosis cases vs no endometriosis**  IRR for more hospital stays  OR for same-day discharge | 0·96 (0·87 – 1·07)  1·24 (1·19 – 1·30) | 0·96 (0·86 – 1·07)  1·27 (1·20 – 1·33) |
|  | **Surgically confirmed cases vs no endometriosis**  IRR for more hospital stays  OR for same-day discharge | 0·88 (0·77 – 1·00)  1·28 (1·22 – 1·35) | 0·87 (0·76 – 0·99)  1·35 (1·27 – 1·43) |
|  | **Clinically suspected cases vs no endometriosis**  IRR for more hospital stays  OR for same-day discharge | 1·13 (0·96 – 1·33)  1·17 (1·10 – 1·26) | 1·13 (0·96 – 1·34)  1·12 (1·03 – 1·21) |
|  | **Only hospital cases vs no endometriosis**  IRR for more hospital stays  OR for same-day discharge | 0·84 (0·74 – 0·96)  1·26 (1·20 – 1·33) | 0·83 (0·73 – 0·95)  1·30 (1·22 – 1·38) |
| IRR = incidence rate ratio; ORs = odds ratios; CI = confidence interval. Women without endometriosis were used as a reference group. To identify all cases of endometriosis, we linked the ALSWH survey responses with three key administrative health databases: MBS, PBS, and the hospital-admitted patient databases. Surgically confirmed cases were identified using records from the MBS or hospital databases. Clinically suspected cases included women who either reported endometriosis in the ALSWH surveys or were prescribed medications specific to endometriosis as indicated by the PBS database. We used only the hospital database of admitted patients up to 2022 to identify hospital cases through ICD-10-AM and ICD-9-CM codes. The incidence rate ratio was adjusted for age at admission, residence, education, and management income.  ^†^ First analysis: The length of stay was determined by calculating the difference between the admission and separation dates and subtracting total leave days. If a patient was admitted and discharged on the same day, the length of stay was recorded as one day. A value of 0 indicated no hospitalisation.  ^‡^ Second analysis: The length of stays was calculated using the same method, but only for hospitalised individuals. Those without any hospitalisations each year were excluded to account for same-day hospitalisations. In this analysis, if admission and discharge occurred on the same day, the length of stay was considered 0 days. | | | |
| **Supplementary Table 4: Incidence Rate Ratio of all-cause hospitalisations and length of stay among women with or without endometriosis, n= 13501 (women with endometriosis= 1963, without endometriosis= 11538)** | | | |

**Supplementary Figure 1:** Hospitalisations by site of endometriosis, 4th character ICD-10-AM code. Endometriosis of the uterus (ICD-10-AM code N80·0) includes adenomyosis. Other endometriosis (N80·8) includes endometriosis of the thorax. We used only the hospital database of admitted patients up to 2022 to identify cases.


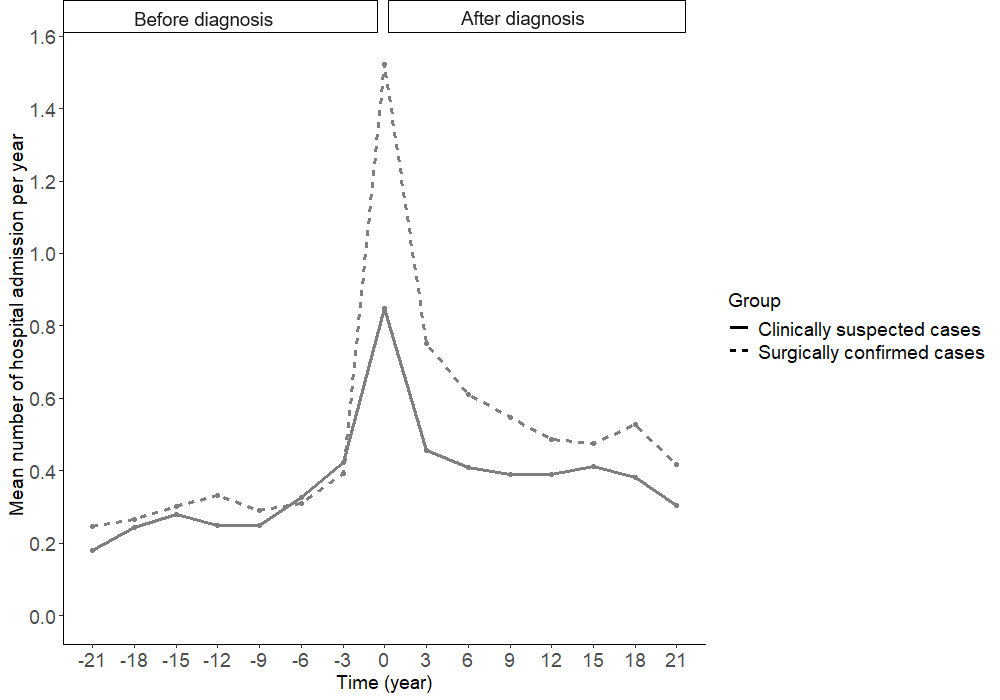


**Supplementary Figure 2:** The mean number of hospital admissions according to time of diagnosis among women with surgically confirmed and clinically suspected endometriosis (n= 1963)


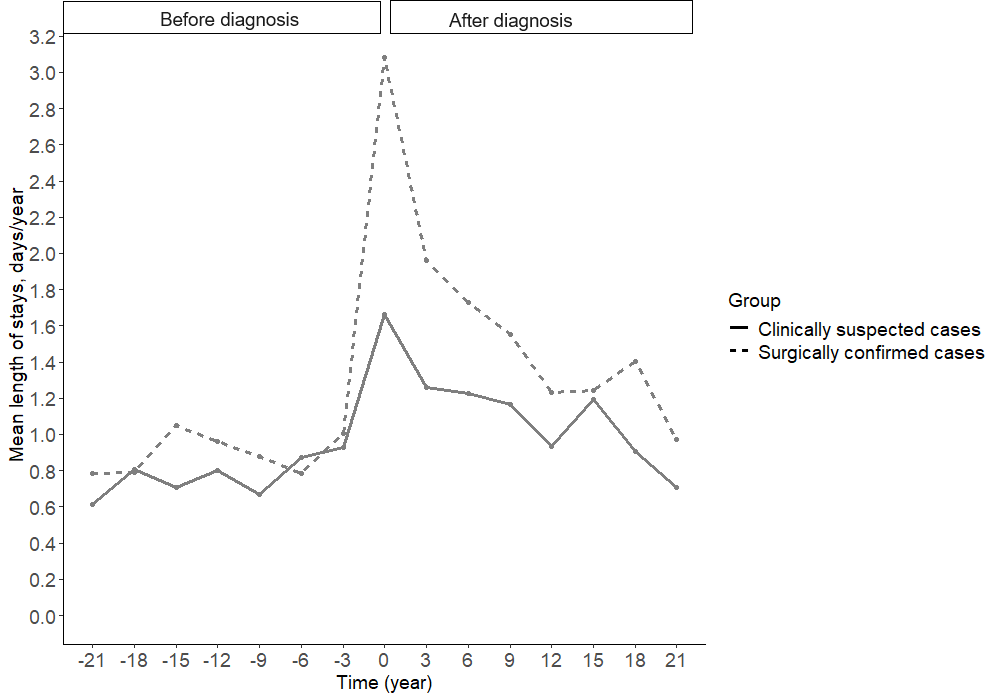


**Supplementary Figure 3:** The mean length of hospital stays according to time of diagnosis among women with surgically confirmed and clinically suspected endometriosis (n= 1963)
